# Field‐Free Spin‐Splitting‐Torque Driven Stochastic Neuron Mimicking the Neuromorphic Imagination for High‐Performance Recognition

**DOI:** 10.1002/advs.75654

**Published:** 2026-05-11

**Authors:** Junwei Zeng, Baoshan Cui, Xi Guo, Jiahao Liu, Xiaoyu Feng, Liang Fang, Li Xi, Xiaolong Fan, Xiaoxi Liu, Yang Guo

**Affiliations:** ^1^ College of Computer National University of Defense Technology Changsha China; ^2^ Key Laboratory of Magnetism and Magnetic Functional Materials Ministry of Education Lanzhou University Lanzhou China; ^3^ College of Advanced Interdisciplinary Studies & Hunan Provincial Key Laboratory of Novel Nano‐Optoelectronic Information Materials and Devices National University of Defense Technology Changsha Hunan China

**Keywords:** artificial neural network, ferromagnetic device, field‐free switching, neuromorphic computing, spin splitting torque

## Abstract

The human brain can construct coherent spatial imagery in the absence of sensory input—an “imagination” capability that complementary metal‐oxide‐semiconductor transistor (CMOS)‐based artificial neural networks (ANNs) struggle to replicate with comparable energy efficiency and architectural compactness. Realizing this function in hardware requires a single spintronic device that simultaneously provides field‐free switching and intrinsic, Gaussian‐distributed stochasticity; yet such devices have not been reported to date. Here, we report field‐free spintronic neuromorphic devices that exploit the spin‐splitting effect of altermagnetic RuO_2_. The resulting stochastic neuron exhibits Gaussian‐distributed outputs, which reduces the number of devices required by about 87%. Benefiting from the high thermal conductivity and micrometer footprint of RuO_2_, transient Joule heating drives stochastic, field‐free magnetization reversal in the adjacent Co/Pt multilayer. Leveraging these devices, we implement an all‐spin ANN to restore CIFAR‐10 images with 50% occlusion. The reconstructed images achieve a high Fréchet Inception Distance score of 1.98 and a classification accuracy of ∼90%, and a 3.75‐fold improvement in recognition performance. Our work establishes an energy‐efficient, hardware‐level pathway toward brain‐inspired imagination systems, advancing the functional emulation of cortical associative processes.

## Graphical abstract

The human brain's imagination, which enables autonomous driving hazard avoidance by completing missing visual information, relies on Gaussian‐stochastic neuron. We report the altermagnetic RuO_2_ spintronic neurons integrating field‐free switching and intrinsic Gaussian stochasticity, building an all‐spin ANN for high‐quality image repairing and high‐accuracy recognition.

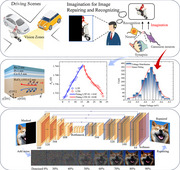

## 1 Introduction

The human brain effortlessly imagines coherent scenes—anticipating a partially occluded object, replaying a memory, or inventing a new visual context—without any concurrent sensory input. This generative faculty arises from recurrent circuits in the hippocampus and visual cortex that exchange probabilistic predictions until uncertainty is minimized and a vivid percept emerges [1, 2]. Artificial neural networks (ANNs) attempt to mimic this process by injecting Gaussian noise into deep generative models [3], but their realization on von‐Neumann/CMOS hardware faces two fundamental bottlenecks: (1) the physical separation of memory and computation inflates energy and latency; (2) dozens of transistors are required to approximate a single stochastic neuron, exploding chip area.

To address these challenges, emerging spintronic devices offer a promising route to mimic the brain's generative imagination. At the core is a stochastic neuron that produces Gaussian‐distributed noise. Spintronic devices exhibit intrinsic, thermally activated random behavior, providing a physical basis for such Gaussian neurons. Recent works have used skyrmion number or size fluctuations to realize a true random number generator [4, 5, 6]. By contrast, approaches that rely on the stochastic switching of a single magnetic tunning junction (MTJ) yield sigmoidal output statistics [7, 8, 9], and multi‐MTJ circuit compositions only approximate a Gaussian distribution [10]; both make it difficult to obtain high‐quality, controllable Gaussian random signals from a single device.

Moreover, most spintronic devices require an external magnetic field during spin‐orbit‐torque (SOT) [11, 12] writing to break symmetry and achieve deterministic switching [13, 14, 15, 16, 17, 18]. This approach hinders high‐density neuromorphic integration and the implementation of brain's imagination. Voltage‐controlled magnetic anisotropy offers a field‐free method, but it faces electrical breakdown risk [18, 19]. The exchange bias of an antiferromagnet provides built‐in symmetry breaking, enabling field‐free switching [20, 21, 22]. However, once the device parameters are set, the exchange‐bias field is fixed and lacks tunability. Likewise, exploiting vertical material gradients to generate a nonuniform SOT acting on the magnetic layer can achieve field‐free switching, but it similarly lacks tunable flexibility [23, 24]. Yet, to date, no single spintronic element has simultaneously delivered (i) deterministic, field‐free writing that eliminates power‐hungry electromagnets, and (ii) programmable, Gaussian‐distributed stochasticity that can serve as both the “neuron” and the “synapse” of a generative network.

The altermagnet [25, 26], such as RuO_2_ [27, 28, 29, 30], produces spin splitting torque (SST) enabling the field‐free switching, compared to a traditional SOT device. Recent reviews and studies have experimentally and theoretically validated the altermagnetic characteristics of RuO_2_ [31, 32, 33, 34]. The spin splitting in its electronic bands enables an applied current to generate a spin current collinear with the antiferromagnetic Néel vector. This mechanism enables unique, field‐free control, allowing the spin current's direction and magnitude to be tuned by reorienting the Néel vector [29]. Moreover, the RuO_2_ with high thermal conductivity [35] and small device size could accelerate the heating accumulation in the ferromagnetic layer. The stochastic switching occurs in the ferromagnet, which is promising for mimicking the stochastic neuron with Gaussian behavior. Toward the hardware implementation of the efficient brain's imagination, the stochastic spintronic devices, as well as field‐free switching that can mimic the function of the imagination are required, which motivates this study.

In this work, we present a field‐free stochastic neuromorphic device that exploits the spin‐splitting effect. The device integrates a stochastic neuron exhibiting a Gaussian output distribution, which reduces the number of devices required by about 87%. A synapse capable of both long‐term potentiation (LTP) and long‐term depression (LTD), essential for synaptic plasticity. To evaluate the associative inference capability, an all‐spin ANN based on these devices is employed to reconstruct CIFAR‐10 images with 50% occlusion. Quantitative assessment of the reconstructed outputs yields a Fréchet Inception Distance (FID) of approximately 1.98, indicative of high perceptual fidelity. Moreover, the reconstructed images achieve a classification accuracy of about 90% and a 3.75‐fold improvement in recognition performance. Collectively, these results substantiate that the proposed ANN exhibits pronounced associative functionality, effectively emulating the imaginative processes attributed to the human brain.

## 2 Device Fabrication and Characterization

**FIGURE 1:**
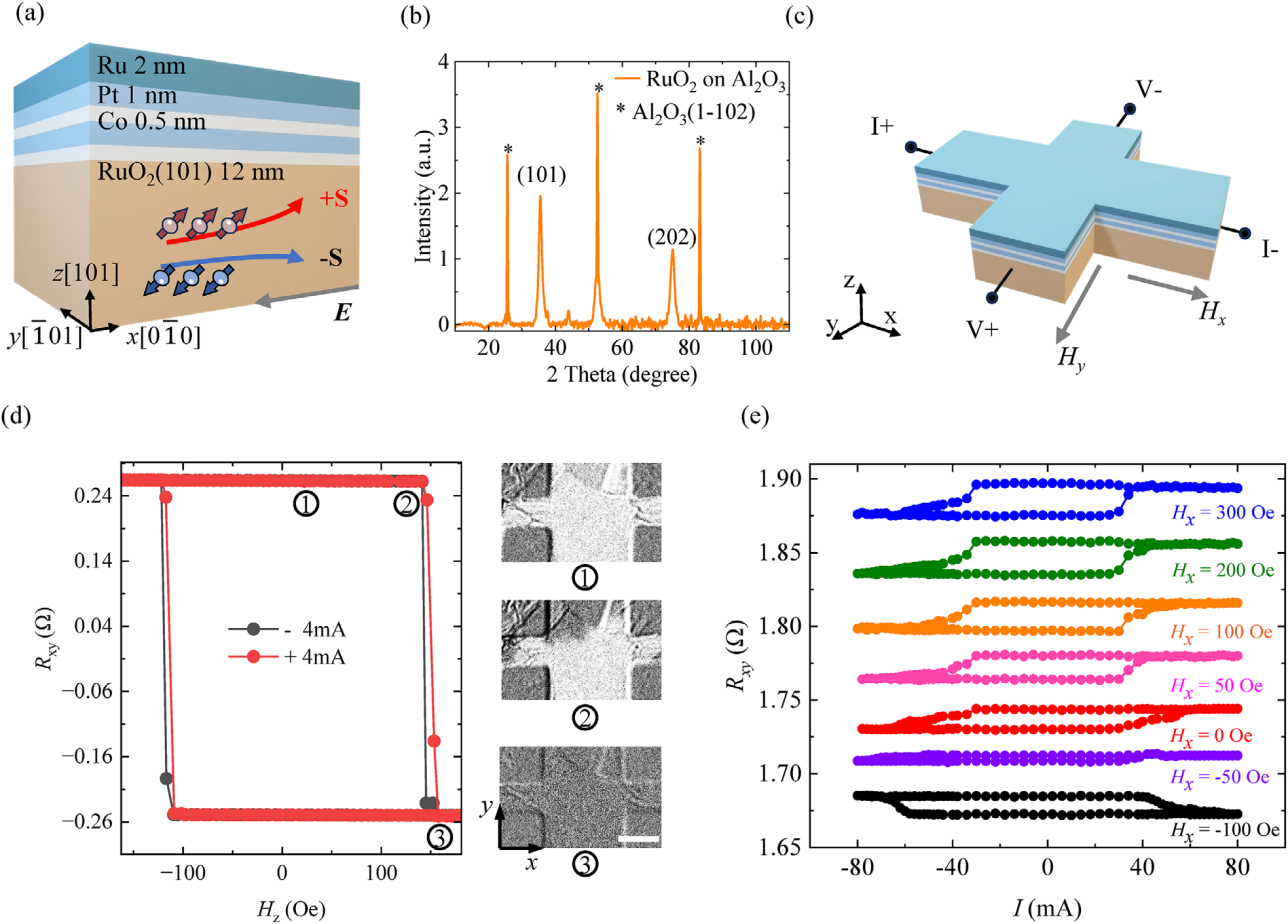
(a) The schematic illustration of the ferromagnetic multilayers. (b) The XRD spectra of RuO_2_. (c) The schematic illustration of the ferromagnetic device. (d) The AHE resistance *R*_xy_ as a function of the perpendicular magnetic field *H*_z_. The magnetic domain configurations at the selected magnetic fields are recorded by using a p‐MOKE imaging microscope. The scale bar is 10 µm. (e) SST switching under a different in‐plane magnetic field *H*_x_ applied colinear to the current direction.

The multilayer of stacking order: RuO_2_(12)/[Co(0.5)/Pt(1)]_2_/Ru(2) (numbers denote the thickness in nanometers) is deposited by using an ultrahigh vacuum magnetron sputtering system on the Al_2_O_3_ substrate. Figure 1a schematically illustrates the magnetic structure of ferromagnetically coupled Co and Pt layers. Figure 1b displays the X‐ray diffraction (XRD) results of the RuO_2_ film. The diffraction spectra exhibit exclusively the (101) and (202) peaks corresponding to RuO_2_, alongside the substrate signal from Al_2_O_3_, there are no impurity phases are detected within the instrumental detection limit. The magnetic multilayer is further patterned into Hall bar devices by using standard photolithography and ion milling, as shown in Figure 1c. The anomalous Hall effect (AHE) is employed to characterize the magnetization states of [Co/Pt]_2_ layers. For the AHE measurements, the currents are applied along the longitudinal direction (*x*‐axis), while the Hall resistances *R*_xy_ are measured along the transverse direction (*y*‐axis). The square‐shapes AHE loops shown in Figure 1d confirm the presence of perpendicular magnetic anisotropy (PMA) of the [Co/Pt]_2_ multilayers. The polar magneto‐optical Kerr effect microscope (p‐MOKE) images suggest that the magnetic field‐driven magnetization switching process is completed by the domain wall nucleation and motion. Furthermore, by applying larger currents of ±4 mA, a clear AHE loop‐shifts can be observed in Figure 1d, predicting the existence of out‐of‐plane effective field, which can enable the field‐free SST switching. Then, the current‐driven SST switching is performed in a Hall bar device: the writing current pulses with a width of 0.1 ms are applied along *x* direction to provide the SST to deterministicly switch the [Co/Pt]_2_ layer under the assistance of an in‐plane magnetic field along *x* direction (*H*_x_). After each writing pulse with a 3 s delay, *R*_xy_ is measured to detect the magnetization state by a small dc reading current of 1 mA. Figure 1e displays the current‐induced SST magnetization switching loops under different *H*_x_ from +300 to –100 Oe. We observed that the magnetization can be efficiently switching by SST, and the switching polarity reverses upon the reversal of the magnetic field direction. In particular, the current‐driven deterministic SST magnetization switching is almost completely suppressed at an in‐plane magnetic field *H*_x_ of −50 Oe. The physical origin of this phenomenon lies in the almost full cancellation between the torques generated by the in‐plane spin polarization *σ*_y_ and the out‐of‐plane spin polarization *σ*_z_ in the system. Significantly, we can clearly see a robust field‐free SST switching, meanwhile, presenting nonvolatile multi‐magnetization states.

**FIGURE 2:**
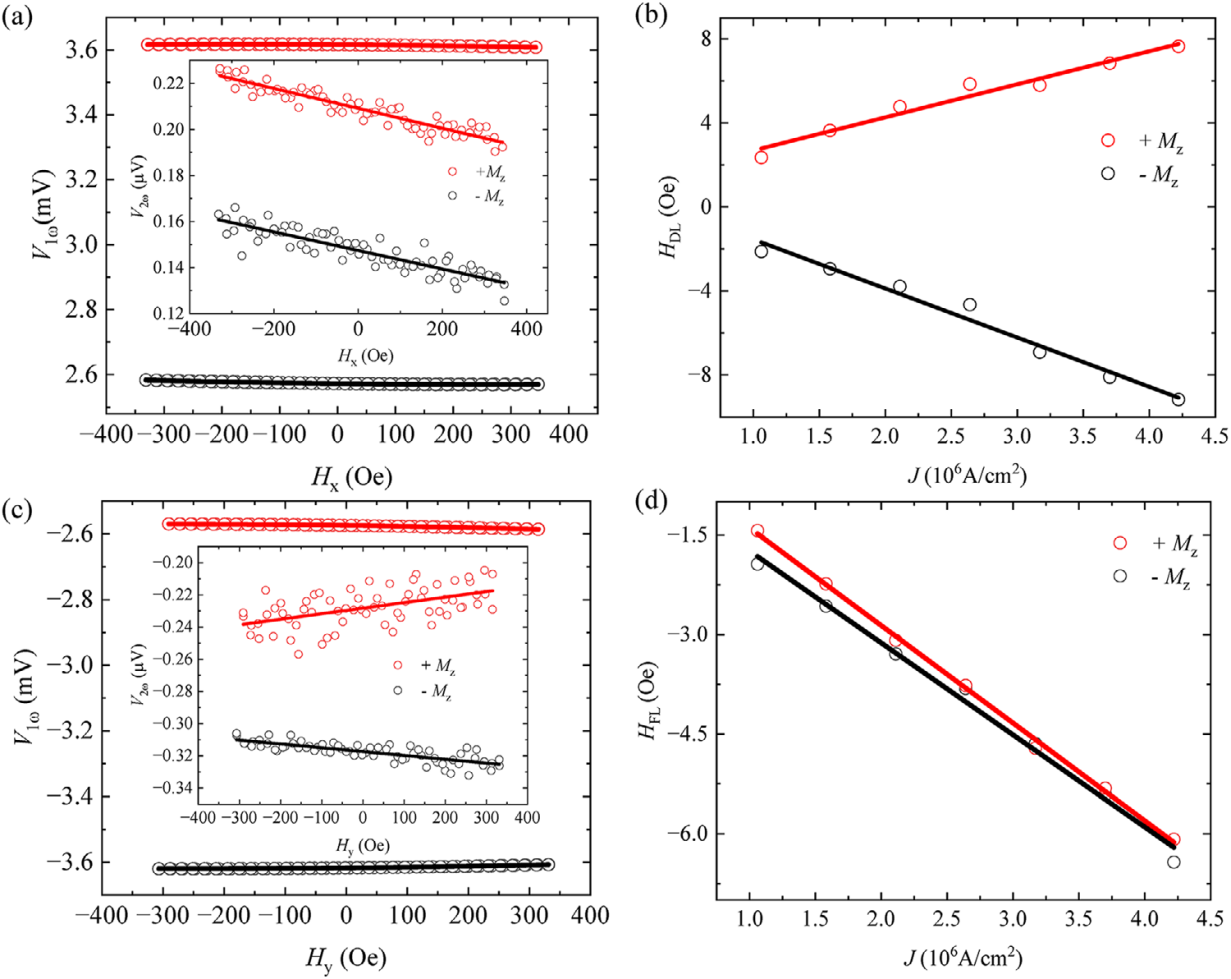
Measurement of SST efficiency. (a, b) Damping‐like effective field: (a) first‐order harmonic component (*V*_1ω_); (b) second‐order harmonic component (*V*_2ω_) (c, d) Field‐like effective field: (c) first‐order harmonic component (*V*_1ω_); (d) second‐order harmonic component (*V*_2ω_).

Next, the second‐harmonic technique is employed to quantified the SST efficiency by injecting an alternating current with a frequency of 133 Hz along the longitudinal channel and sweeping the in‐plane magnetic field along the longitudinal direction. Simultaneously, the first harmonic (*V*_1ω_) and the second harmonic (*V*_2ω_) Hall voltages are measured with two lock‐in amplifiers. Figure 2a–c shows the dependence of the first and second harmonic results with the external field along the longitudinal direction (*H*_x_)/transverse direction (*H*_y_). Then, the damping‐like (*H*_DL_) (Figure 2b) / field‐like (*H*_FL_) (Figure 2d) effective fields upon different current densities can be calculated with $H_{DL}=-2\frac{\partial V_{2\omega }}{\partial H_{x}}/\frac{\partial^{2}V_{1\omega }}{\partial H_{x}^{2}}\left(H_{FL}=-2\frac{\partial V_{2\omega }}{\partial H_{y}}/\frac{\partial^{2}V_{1\omega }}{\partial H_{y}^{2}}\right)$, where the numerator and denominator are fitted by the first and second harmonic signals with the parabolic and the linear function, respectively. The effective spin hall angle is about 0.07 (The details are shown in the Part S1, including the field‐like component and damping‐like component). The value of the effective spin hall angle indicates an efficient spin‐to‐charge conversion derived from the SSE in the heterostructure.

## 3 Results and Discussion

### 3.1 Synapse

**FIGURE 3:**
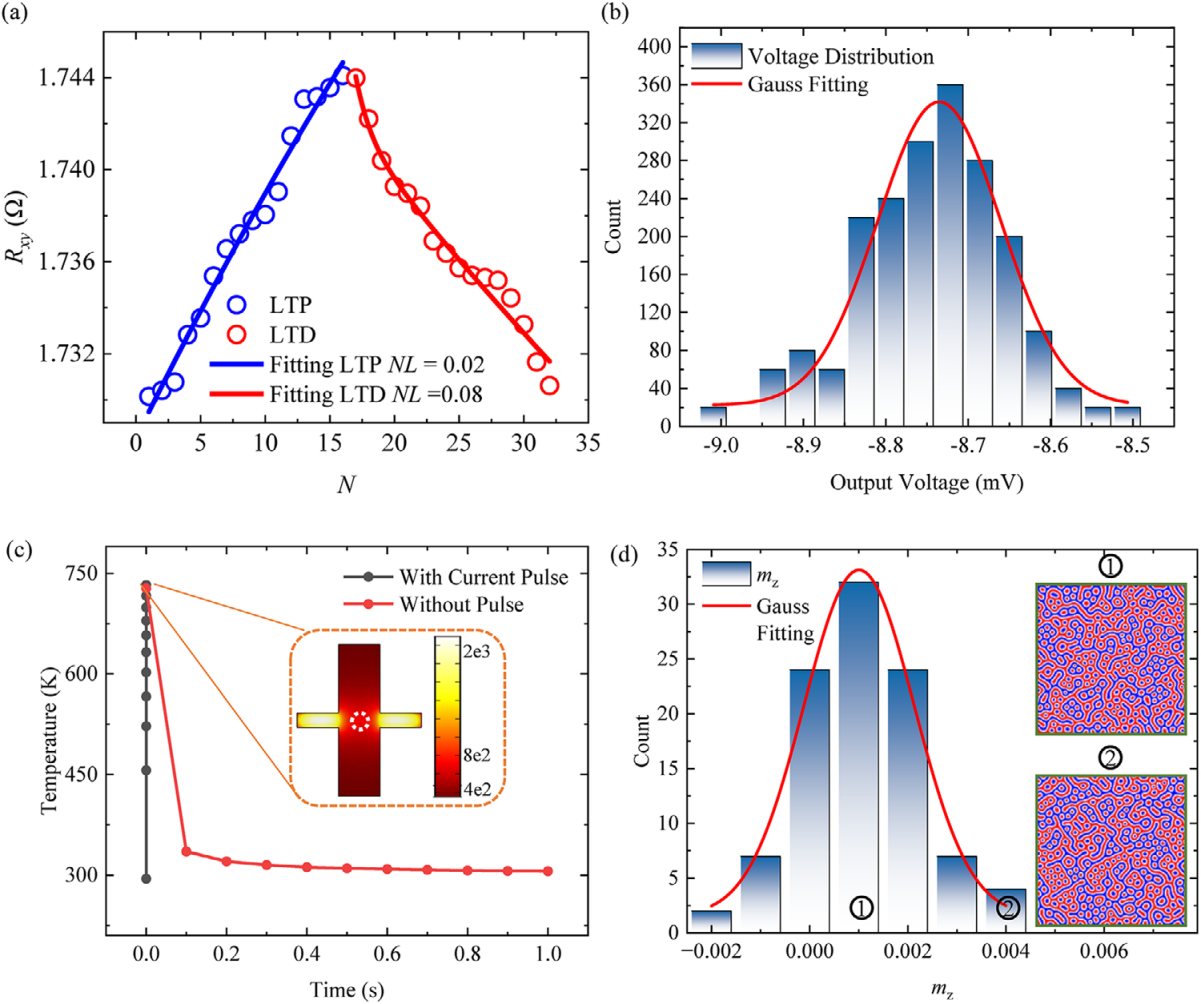
(a) The demonstration of LTP and LTD is enabled by increasing the number of current pulses. (b) The stochastic neuron output behaviors. (c) Finite element method simulation showing the variation in device temperature under the pulse current excitation. (d) The Mumax3 simulates thermal‐assisted SST switching results at 732 K.

As mentioned above, the SST‐driven efficient magnetization switching exhibits non‐volatile multilevel states, which can effectively emulate prominent synaptic behaviors with long‐term memory. The synapse is located at junctions between pre‐ and post‐synaptic neurons, with the function of dynamically adjusting the connection strength (namely, synaptic weight) between the neurons. In an artificial synapse, the synaptic weight can be mimicked by the amplitude of *R*_xy_ depends on the magnetization direction of the Co. of which results are shown in Figure S1b of Part S2. As shown in Figure 3a, a sequence of 16 consecutive positive (negative) pulses is employed to achieve the LTP (LTD) process by increasing (decreasing) the amplitude of the current pulses with a range of 30 to 45 mA. The nonlinearity (*NL*) of the LTP/LTD is ≈0.02 and 0.08, which reflects the better linear resistance change by the current‐induced SST (the equation of *NL* is proposed to describe the synaptic linearity, which is shown in Part S3). This linear resistance change induced by current‐driven SST is essential for achieving high precision and stability in neuromorphic computing systems. Figure S1a shows low device‐to‐device variation and cycle‐to‐cycle variation in the three‐cycle testing.

The reported spintronic synaptic devices mainly adopt two pulse modulation schemes: constant‐amplitude pulses (identical pulse schemes in Part S4) and amplitude‐incremental pulses. As shown in Table 1, devices adopting the amplitude‐incremental pulse scheme exhibit significantly higher linearity than those using identical pulse schemes. The high nonlinearity of identical‐pulse devices originates from inhomogeneous pinning in the magnetic thin film: under fixed‐amplitude pulses, low‐pinning regions undergo preferential magnetization switching and are gradually depleted, while the fixed spin torque cannot overcome high‐pinning energy barriers, shrinking the single‐pulse switching area and decaying Δ*R*. Furthermore, the two schemes have been implemented to realize field‐free switching. Notably, altermagnets represented by RuO_2_ exhibit unique advantages for this application, including simultaneously high spin diffusion length and spin‐charge conversion efficiency, as well as spin splitting that can be modulated via multi‐dimensional low‐power means including strain, electric field, light, and heat.

**TABLE 1:** The comparison between the synapse designs.

Magnetic Materials	Write Mechanism	Write Current	Synapse *NL*_LTP/LTD_	Pulse Schemes	Weight States	Field‐free Switching	Cite
DW‐MTJ	SOT	0.2 V	Tunable	Increasing magnitude	5	Yes	[40]
Ti/CoFeB/Ta	SOT	1e11 A/m^2^	0.01/0.29	Identical	20	*H*_x_ = 50 Oe	[41]
Pt/CoFeB/MgO	SOT	2.57e11 A/m^2^	0.01/0.01	Increasing magnitude	35	*H*_x_ = 40 Oe	[42]
PtMn/Co/Ni/Co	SOT	10.5V	0.5/1.44	Identical	100	Yes	[43]
Pt/Co/SiO_2_	SOT	2e11 A/m^2^	0.08/0.09	Identical	80	*H*_x_ = 300 Oe	[44]
Ta/Co/Ta	ED	1 V	3.2/2.91	Identical	30	Yes	[45]
Ta/Pt/Co/Pt	SOT	3.03e11 A/m^2^	3.48/8.96	Identical	30	*H*_x_ = 50 Oe	[46]
W/CoFeB/MgO	SOT	∼1e6 A/m^2^	—	Identical	9	*H*_z_ ≤ 10 Oe	[47]
WTe_2_/FeGeTe	SOT	4.2 mA	—	Increasing magnitude	8	Yes	[48]
EB‐MTJ	SOT	1.8 V	0.01/0.01	Increasing magnitude	30	Yes	[49]
**This work**	SST	2.2e11 A/m^2^	0.01/0.08	Increasing magnitude	16	Yes	

### 3.2 Stochastic Neuron

Biological neurons exhibit probabilistic behavior, characterized by fluctuations in the membrane potential caused by stochastic ionic currents and intrinsic cellular noise. In particular, thermal noise arising from random ion diffusion and probabilistic channel gating contributes significantly to neuronal output variability. These stochastic fluctuations facilitate diverse associative responses across varying contexts, thereby enhancing the flexibility and adaptability of neural processing. In Figure 3b, we applied a bipolar pulse sequence to the device: an 80 mA negative reset pulse (with a width of 100 µs), followed 6 s later by a 42 mA positive write pulse (with a width of 100 µs), and finally, a 5 mA read pulse was applied 3 s after the write pulse. This read pulse was used to measure the anomalous Hall voltage. The 2000‐cycle operation yielded output voltages that conformed to a Gaussian distribution, as confirmed by the quantile‐quantile plot in Figure S3 (Part S5 explains the stochastic behavior by the LLG equation [36]). The AHE voltage of the device is directly correlated with the out‐of‐plane magnetization *m*_z_ of the magnetic domains, meaning the observed Gaussian voltage distribution directly reflects the statistical distribution of the device's internal *m*_z_ states. Elevated current amplitudes induced significant Joule heating, which promoted stochastic switching behavior. Finite element simulations were employed to quantify this thermal effect. As depicted in Figure 3c, the baseline device temperature remained stable at 293 K in the absence of writing current. In contrast, the application of a pulse resulted in rapid heating to 732 K within the 100 µs pulse duration. Subsequent cooling reduced the temperature to 306 K within 1 s. The 6‐second interval between reset and write pulses exceeded this cooldown period, ensuring complete dissipation of the thermal energy generated by the reset pulse prior to the initiation of the write operation. Micromagnetic simulations performed using Mumax3 further confirmed that write‐pulse heating enhances stochastic switching behavior. As illustrated in Figure 3d, the resulting magnetic domains are randomly distributed and exhibit characteristics consistent with a Gaussian distribution. These collective findings indicate that the Hall bar devices inherently operate as stochastic neurons, thereby implementing neuromorphic functions that parallel the associative image generation processes observed in the human brain, while also reducing the number of required devices by about 87%.

### 3.3 All‐Spin ANN for Mimicking Imagination

**FIGURE 4:**
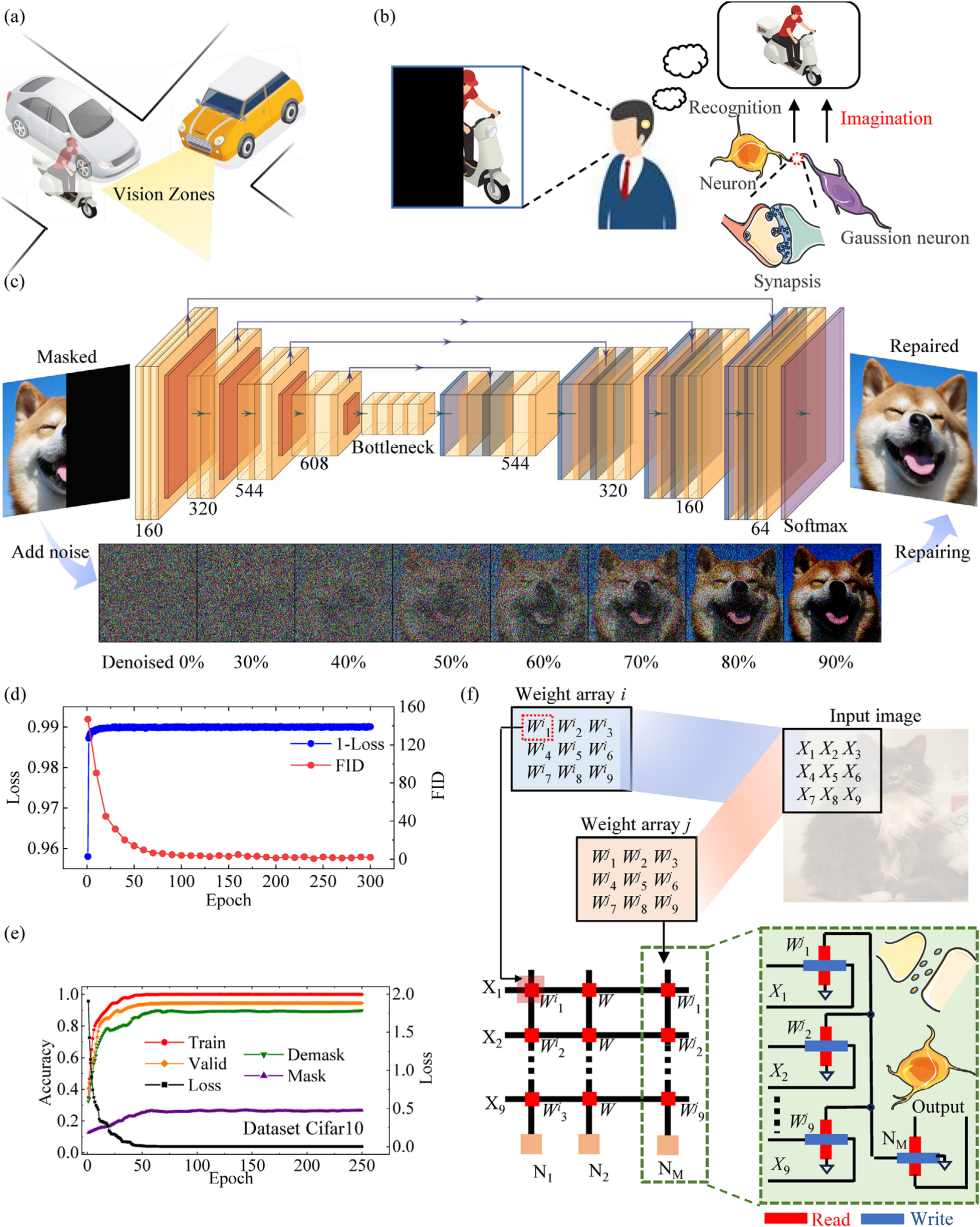
(a) The driving scenarios of the real world. (b) The process of the human imagination through the intertwined neural networks concluding neurons and synapses. (c) The architecture of the proposed ANN to mimic human imagination for image inpainting. (d) The FID score and loss as a function of training epoch. (e) The schematic of the synaptic array architecture for the convolution and activation.

We integrate the proposed SST‐driven synapses and stochastic neuron into an all‐spin ANN to emulate the imagination capability of the human brain, achieving accurate image inpainting for high recognition performance. In real‐world driving scenarios, as illustrated in Figure 4a, a scooter may suddenly appear from behind a white car, thereby obstructing the view of a yellow car positioned ahead on the right. Due to the occlusion, the scooter becomes detectable only when it partially emerges from behind the white car. The human brain, however, can employ associative reasoning to anticipate the presence of the scooter; nevertheless, delayed reaction times may still lead to accidents. In such situations, the missing visual information hinders the vehicle's neural network from accurately detecting the scooter. If the vehicle's system could employ brain‐like associative functions, specifically using image inpainting to reconstruct missing details, it could enable faster and more accurate detection, potentially preventing accidents.

Herein, we propose an all‐spin ANN designed to computationally emulate the brain's cortical mechanisms for generative imagination. The architecture is based on a U‐Net‐based diffusion model, which initializes occluded regions with stochastically generated Gaussian noise and progressively refines the image over 1000 iterative denoising steps. As shown in Part S6, the system employs principles derived from spintronic devices to simulate the brain's associative functions, providing an accurate restoration of missing image content (The Part S7 demonstrates the pathway to reduce the iteration count from algorithmic optimization and device‐level optimization). Trained on the CIFAR‐10 dataset and evaluated via FID metrics, our framework achieves a high FID score of 1.98 after 100 training epochs, indicating that the model converges to near‐photorealistic generation quality. As demonstrated in Figure 4c, using a half‐occluded dog image, each denoising iteration enforces multi‐scale semantic consistency through classifier‐free guidance, ultimately producing a complete visual restoration after 1000 refinements.

Quantitative evaluation reveals a dramatic improvement in recognition accuracy: 94% for original images, 24% for occluded inputs (50% masking), and 90% for reconstructed images. This 275% accuracy recovery empirically demonstrates the efficacy of the proposed neuromorphic framework in translating biological associative processes into artificial image reconstruction capabilities. Driven by spintronic synapses and stochastic neurons, this framework introduces a brain‐inspired methodology for dynamic scene reconstruction. It advances the frontiers of visual perception and pattern recognition in autonomous systems, particularly in environments where missing or incomplete information is prevalent. The successful integration of spintronic principles into ANNs for image inpainting underscores the potential for neuromorphic systems to simulate human‐like imagination and associative reasoning. By harnessing thermal fluctuations and stochastic behaviors of spintronic devices, our system mimics the capacity of the brain to infer missing sensory information, rendering it particularly suitable for dynamic and unpredictable real‐world applications, including autonomous driving and augmented reality. To further clarify the advantages of our work, Table S1 of Part S8 demonstrates the comparisons between our generative performance and both software platforms and neuromorphic devices. Most emerging neuromorphic generative devices adopt voltage‐modulated stochastic writing to mimic Sigmoid random noise for RBM‐based generation tasks [37, 38, 39], while the inherent shallow two‐layer structure of RBM cannot support deep feature fitting, and is only applicable to simple low‐dimensional data generation, failing in complex practical scenarios. The diffusion model [3] proposed in 2020 fundamentally overcomes RBM's drawbacks via deep architectures like U‐Net, with far stronger generation capability for high‐dimensional complex data. In 2025, University of California researchers realized hardware diffusion generation with VCMA‐MRAM emulating Gaussian noise [10], but this solution has excessive device overhead. Our work achieves two core improvements: 87% total device count reduction over the VCMA‐MRAM scheme, and ∼1.60× generation performance improvement over the software baseline (FID score reduced from 3.17 [3] to 1.98). These developments represent an initial stride toward the realization of neuromorphic computing systems capable of adaptive and fault‐tolerant learning akin to that observed in the human brain.

## 4 Conclusions

In conclusion, we have demonstrated an all‐spin ANN built using the proposed field‐free, stochastic neuromorphic devices driven by SST. These devices integrate Gaussian‐distributed stochastic neurons and synapses, supporting both LTP and LTD. The system effectively reconstructs images with significant occlusion. The restored CIFAR‐10 images achieve a FID of approximately 1.98 and a classification accuracy of about 90%, jointly evidencing high perceptual fidelity and semantic preservation. These findings confirm that the ANN exhibits pronounced associative functionality, providing a hardware‐based emulation of the imaginative capabilities characteristic of the human brain.

## 5 Method

### 5.1 Experiment

Thin films were deposited sequentially from bottom to top in a chamber with a base pressure of 2 × 10^−^^8^ Torr. The substrate was resistively heated to 550°C under 20 sccm Ar/5 sccm O_2_ atmosphere maintained at 0.5 mTorr, stabilized for 20 min followed by 5‐min target pre‐sputtering at 25 W with the shutter closed. RuO_2_ was then deposited at 25 W (5 × 10^−^^4^ Torr) with thickness controlled by the calibrated growth rate of 0.0343 nm/s, followed by in situ high‐vacuum annealing (<1 × 10^−^^7^ Torr) at 550°C for 20 min. After cooling to room temperature under vacuum, the vertical magnetic layer ([Co (0.5 nm)/Pt (1 nm)]_2_) and Ru capping layer were deposited in position. The magnetic multilayer film was patterned into a Hall‐bar device by using standard photolithography and ion milling.

The electrical transport property measurements are carried out by using a Keithley 6221 current source and a Keithley 2182A. The second harmonic measurement was applied by using a Keithley 6221 current source and two Stanford Research SR830 lock‐in amplifiers. The magnetization configuration images were taken by a p‐MOKE imaging microscope.

### 5.2 Simulation

The behavioral functions of the spintronic synapse and stochastic neuron are derived from fitting of the experimental measurement data. The spintronic synapse model serves as the weight arrays for both the diffusion model and the ResNet‐18 network for CIFAR‐10 image classification, while the stochastic neuron model is exclusively implemented in the Gaussian noise generator of the diffusion model. Furthermore, the simulation details have been supplemented in the Methods section of the revised manuscript to enable full reproducibility of our network‐level simulations.

#### 5.2.1 Neuromorphic Imagination Training

The image generation simulations were performed using the CIFAR‐10 dataset, with all input 3‐channel RGB images preprocessed to a fixed 64 × 64 resolution via random resized cropping, horizontal flipping, and normalization to the [‐1, 1] range. The generation task was implemented with a classifier‐free guided denoising diffusion probabilistic model, configured with a 1000‐step noise schedule where the variance β increases linearly from 1e‐4 to 0.02. The denoising network is a U‐Net architecture with an encoder‐decoder channel configuration of [32, 64, 128, 256], 2 residual blocks per resolution level, AdaNorm for time and class conditional embedding modulation, and multi‐head self‐attention modules at the 256‐channel feature level, with a fixed 1024‐dimensional embedding for both sinusoidal time positional encoding and 10‐class conditional embedding. The simulation was trained up to 300 epochs. The training was performed using the AdamW optimizer with a mean squared error loss function between predicted and actual noise. The initial learning rate was set at 3e‐4, which decayed exponentially with a gamma factor of 0.991 per epoch, with a batch size of 140.

#### 5.2.2 Image Repairing

No random cropping or horizontal flipping was applied during evaluation to ensure deterministic processing, and images were normalized to the [‐1, 1] range. Binary half masks were used to define inpainting regions, specifying ground‐truth pixels to retain. The inpainting task was implemented with a trained and classifier‐guided denoising diffusion probabilistic model. The model operated in 16‐bit floating‐point for inference efficiency, with a dedicated classifier guiding the sampling process at a scale of 1.0. During inference, timestep respacing was set to 250 steps for acceleration, paired with a jump length of 10 to iteratively refine the inpainted regions. The inpainting injection schedule was enabled to properly inject ground‐truth pixels into the sampling process.

#### 5.2.3 Image Classification

Image classification simulations of the CIFAR‐10 datasets were performed. The offline training simulation was conducted for up to 250 epochs. The demasked/masked/original CIFAR‐10 datasets consist of 50 000 training images and 10 000 testing images, with a batch size of 512. For each training iteration, the cross‐entropy loss function was used, and the weights were updated using stochastic gradient descent with momentum (0.9) and weight decay (5e‐4). The learning rate for the entire model was set to 0.003. During training, random horizontal flips and random cropping (32 × 32 with padding 4) were applied to the input images as part of the data augmentation process. All weights were randomly initialized, and the optimizer updated each synapse according to the computed gradient and learning rate, optimizing the classification accuracy over the CIFAR‐10 validation set.

#### 5.2.4 Finite Element Simulation

A full‐scale (1:1) finite element model was developed to accurately represent the device structure. The model comprises the substrate, a ruthenium (Ru) layer, the various layers constituting the Crossbar device, and the surrounding air medium. To capture the coupled electromagnetic, thermal, and electrical phenomena, we employed a combination of the electromagnetic‐thermal module, the solid thermal conduction module, and the current module. The simulation was performed using a transient solver to accurately determine the dynamic behavior of the device under operational conditions. The key material parameters defined in the model are as follows: for the RuO_2_ layer, the electrical conductivity set to 3 × 10^4^ S/m, thickness set to 12 nm, and the thermal conductivity is set to 50 [W/(m·K)]. For Co/Pt/Ru single layers: thicknesses of 0.5/1/2 nm, thermal conductivities of 100/72/117 W/(m·K), and electrical conductivities of 1.6 × 10^6^ /9.5 × 10^6^ /1.4 × 10^7^ S/m; The electrical conductivity of air was set to 1 × 10^−14^ S/m.

## Author Contributions

J.Z., B.C., X.G., and J.L. contributed equally to this work. J.Z. performed the AHE measurement, neuromorphic device measurement, Mumax3 simulation and the ANN simulation under the guide of L.F. and Y.G. Y.F. prepared the multilayer films and XRD measurement under the guide of X.F. X.G. prepared the devices and the second harmonic measurement under the guide of B.S. and L.X. J.L. performed the finite element simulation. X.F., L.X., L.F., and Y.G. provided financial support.

## Conflicts of Interest

The authors declare no conflicts of interest.

## Supporting information




**Supporting File**: advs75654‐sup‐0001‐SuppMat.docx.

## Data Availability

The data that support the findings of this study are available from the corresponding author upon reasonable request.
