# Supplementary material for: Field‐Free Spin‐Splitting‐Torque Driven Stochastic Neuron Mimicking the Neuromorphic Imagination for High‐Performance Recognition
Source: Adv Sci (Weinh). 2026 May 11;13(44):e75654. doi: 10.1002/advs.75654 (PMC13335861; doi:10.1002/advs.75654)
Supplement: Supplementary file 1 [file ADVS-13-e75654-s001.docx]

1. **Supplementary Information of “Field-free Spin-splitting-torque Driven Stochastic Neuron Mimicking the Neuromorphic Imagination for High-performance Recognition”**

Junwei Zeng^1,†^, Baoshan Cui^2, †,*^, Xi Guo^2,†^, Jiahao Liu^3,†^, Xiaoyu Feng^2^, Liang Fang^1^, Li Xi^2^, Xiaolong Fan^2,*^, Xiaoxi Liu ^2,*^, and Yang Guo^1,*^

*^1^ College of Computer, National University of Defense Technology, Changsha, China, 410073*

*^2^ Key Laboratory of Magnetism and Magnetic Functional Materials (Lanzhou University), Ministry of Education, Lanzhou, China, 730000*

*^3^ College of Advanced Interdisciplinary Studies & Hunan Provincial Key Laboratory of Novel Nano-Optoelectronic Information Materials and Devices, National University of Defense Technology, Changsha, Hunan, China, 410073*

† These authors contributed equally to this work.

Correspondence should be addressed to:

* Baoshan Cui, email: cuibs@lzu.edu.cn

* Xiaolong Fan, email: fanxiaolong@lzu.edu.cn

* Xiaoxi Liu: liuxiaoxi@lzu.edu.cn

* Yang Guo, email: guoyang@nudt.edu.cn

The following parts are to be discussed in the supplementary Information.

Part 1: Characterization of spin split torque.

Part 2: The non-validate test, device-to-device and cycle-to-cycle variation of the synapses.

Part 3: The equation of fitting the synaptic behavior.

Part 4 The fixed-pulse scheme implementing LTP and LTD.

Part 5: The validation of Gaussian distribution by the quantile-quantile plot.

Part 6: The deducing of the imagination based on the spintronic device.

Part 7: The pathway to reduce the iteration count in the imagination based on the spintronic device.

Part 8: The comparisons between our generative performance and both software platforms and neuromorphic devices.

Part 1: Characterization of spin split torque.

The effective spin Hall angle quantifies the charge-to-spin conversion efficiency based on the spin split torque (SST), which also contributes to the magnetization switching. Subsequently, we study the SST efficiency in the crossbar. The second-harmonic technique is employed to determine the effective spin-orbit fields in the samples by injecting an alternating current with a frequency of 133 Hz along the longitudinal channel and sweeping in-plane magnetic field along the longitudinal/transverse direction. Simultaneously, the first harmonic (*V*_1ω_) and the second harmonic (*V*_2ω_) Hall voltages in the transverse channel are measured with two lock-in amplifiers. The dependence of the first and second harmonic results with the external field along the longitudinal direction (*H*_x_)/ transverse direction (*H*_y_) for the device. Then, the damping-like (Δμ_0_*H*_x_)/ field-like (Δμ_0_*H*_y_) effective fields upon different current densities can be calculated with Δ${\mu_{0}H}_{x}(\Delta{\mu_{0}H}_{y})\text{ = -2 }\frac{\partial V_{2\omega}}{\partial H_{x}}/\frac{\partial^{2}V_{2\omega}}{\partial{H^{2}}_{x}}$ , where the numerator and denominator are fitted by the first and second harmonic signals with the parabolic and the linear function, respectively. The effective spin Hall angle could be expressed as:

Where, *θ*_eff_ is effective spin Hall angle. *e* is amount of electronic charge. *t*_FM_ is the thickness of the ferromagnetic layer Co. is reduce Planck's constant. The *J*_e_ is the current density in RuO_2_ layer. Using the parallel resistance model, it can be calculated that the current density flowing through the RuO_2_ is only 45% of the total current density. The calculation process is detailed in the equation. The resistivities *R*_RuO2_, *R*_Ru_, *R*_Co_ and *R*_Pt_ are 265.71 Ω, 3068.57 Ω, 569.14 Ω and 378.57 Ω, respectively.

$\frac{I_{RuO_{2}}}{I_{Total}}=\frac{\frac{1}{R_{RuO_{2}}}}{\frac{1}{R_{RuO_{2}}}+\frac{1}{R_{Ru}}+\frac{1}{R_{Co}}+\frac{1}{R_{Pt}}}=\frac{0.00376307 }{0.00848478}=0.44358$

Based on the experimental data, linear and quadratic functions were adopted to fit the first- harmonic and second-harmonic signals, from which the damping-like effective field *H*_DL_ and field-like effective field *H*_FL_ at different current densities were extracted. The overall effective spin Hall angle was calculated to be approximately 0.07. Further analysis shows that the spin Hall angle contributed by the field-like component is 0.05, while that of the damping-like component is 0.07. Their ratio is about 0.71, indicating that the damping-like contribution is 28.6% larger than the field-like contribution (*H*_DL_/*H*_FL_ ≈1.4). These quantitative results confirm that the spin splitting effect originates predominantly from the bulk contribution of RuO₂ rather than the interfacial effect, which physically clarifies and strongly supports the deterministic field-free magnetization switching behavior of the device.

Part 2: The non-validate test, device-to-device and cycle-to-cycle variation of the synapses.

Figure S1. (a) The CTC and DTD variation of the synapse device. (b) the retention properties of the multilevel states.

Figure S1(a) has shown low CTC/DTD variation in the three cycles testing. Figure R1(b) characterizes the non-volatile performance of the synaptic devices. Figure S1 (b) demonstrates the non-volatility of the synapses. The seven states are adjusted by the different amplitude of current pulse. After applying the write pulse, the 50 read currents with 1 mA (*I*_read_ < *I*_write_threshold_) and 20s pulse interval are used to detect the synaptic state. The results of Figure S1 show the states can be maintained with lower fluctuation (the standard deviation σ keeps 2.2e-4 to 2.8e-3).

Part 3: The equation nonlinearity of fitting the synaptic behavior.

The LTP/LTD behavior mode can be described by the following equations

$$\begin{matrix} W_{LTP}=B_{P}(1-e^{-\frac{N}{A_{P}}})+W_{MIN} \\ W_{LTD}=-B_{D}(1-e^{-\frac{N-N_{MAX}}{A_{D}}})+W_{MAX} \\ B_{P,D}=(W_{MAX}-W_{MIN})/(1-e^{-\frac{N-N_{MAX}}{A_{D}}}) \\ \end{matrix}$$

Where *N* and *N*_MAX_ are the numbers of applied pulses and maximum number of applied pulses, respectively. *A*_P_ and *A*_D_ are parameters that represent the nonlinear degree of LTP and LTD, which determine the fitting constant *B*_P_ and *B*_D_.

Part 4 The fixed-pulse scheme implementing LTP and LTD.

Figure S2 The nonlinearity of the synapses. The demonstration of synaptic potentiation and depression that is enabled by the number of current pulses without step current (a constant amplitude of 36 mA).

For the increasing amplitude of the current pulses scheme of Figure 3a, the amplitude gradient of these pulses is precisely controlled by a counter combined with a digital-to-analog converter (DAC). Specifically, the counter accumulates the input pulse number in real time, and its binary counting value serves as the digital input code of the DAC. The analog output of the DAC increases linearly with the counting value, so the amplitude of each subsequent current pulse rises by a fixed step. By configuring the modulus of the counter and the reference voltage of the DAC, we realize a 16-step amplitude sequence ranging from 30 mA to 45 mA for the LTP/LTD characterization.

However, the scheme with variable pulse amplitude achieves superior linearity, simultaneous adjustment of pulse quantity and amplitude will significantly increase the complexity of peripheral driving circuits. When integrated into large-scale neuromorphic crossbar arrays, this design leads to redundant circuit layout, higher power consumption and difficult wiring layout, which greatly restricts its practical deployment.

Thus, we provide the fixed-pulse scheme implementing LTP and LTD, shown in Figure S2. This fixed-pulse scheme shows a relatively higher nonlinearity (*NL_L_*_TP_ = 0.05, *NL_L_*_TD_ = 0.07).

To address this bottleneck, our follow-up research will focus on material engineering optimization of the heterostructure. This optimization is expected to improve the LTP/LTD linearity under fixed single-amplitude pulse excitation, eliminating the need for amplitude adjustment. Ultimately, the device can fully meet the circuit integration requirements of large-scale practical neuromorphic crossbar arrays.

Part 5 The validation of Gaussian distribution by the quantile-quantile plot.

Figure S3. The quantile-quantile plot demonstrates the output distribution of the stochastic neuron.

The normality assumption was assessed using a quantile-quantile (Q-Q) plot. Visual inspection revealed that the sample quantiles closely adhered to the theoretical quantiles of a Gaussian distribution, with data points forming a near-linear alignment along the reference line. Critically, all observations remained within the 95% confidence bands throughout the distribution, indicating no significant deviations in the tails. This graphical evidence strongly suggests that the data follow a Gaussian distribution. The Gaussian function used for fitting is defined as:

$$y=y_{0}+\frac{A}{\sqrt{\pi/2}}e^{-2(\frac{x-x_{c}}{w})^{2}}$$

In Figure 3b (experiment), the baseline *y*_0_ = 1.119, width parameter *w* = 0.150, amplitude *A* = 2.996 and peak center *x*_c_ = −8.735 In the Figure 3d (simulation), the baseline *y*_0_ = 1.575, width parameter *w* = 0.0022, amplitude *A* = 0.0890 and peak center *x*_c_ = 0.001.

This stochastic magnetization switching behavior can be quantitatively explained based on the Landau-Lifshitz-Gilbert (LLG) equation framework. The magnetization dynamics at zero temperature is governed by the conventional Landau-Lifshitz-Gilbert (LLG):

$$\vec{m}=-\frac{\gamma}{1+\alpha^{2}}(m\times B_{eff}+\alpha m\times(m\times B_{eff}))$$

where *m* denotes the normalized magnetization vector, *γ* is the gyromagnetic ratio, and *α* represents the Gilbert damping constant. In this framework, the magnetization evolution is fully determined by the effective field *B*_eff_ and damping term, showing purely deterministic characteristics.

$$\vec{m}=-\frac{\gamma}{1+\alpha^{2}}(m\times(B_{eff}+B_{therm})+\alpha m\times(m\times(B_{eff}+B_{therm})))$$

At finite room temperature, the magnetic system is inevitably affected by intrinsic thermal fluctuations. According to the fluctuation-dissipation theorem^[1]^, lattice thermal vibration can be equivalent to a random thermal effective field *B*_therm_ with a zero mean and intrinsic Gaussian distribution.

Part 6 The deducing of the imagination based on the spintronic device.

The imagination of brain is a class of generative models whose core idea involves reconstructing a target data distribution through iterative denoising, starting from pure noise. The derivation of diffusion models can be divided into two key processes:

1.*Forward Adding Noise Process*: Gradually corrupts the data by adding noise over multiple steps.

2.*Reverse Generative Process*: Learns to iteratively denoise and recover the original data distribution.

Below is the detailed derivation of diffusion models:

1. Forward Adding Noise Process

In the forward process, we gradually add noise to the data samples $x_{0}\sim q(x_{0})$over multiple timesteps until they converge to a noise-like distribution (typically isotropic Gaussian). Let $q(x_{t} \text{∣}x_{t-1} )$denote the conditional distribution at step *t*. The forward process is defined as follows:

Where, $\beta_{t} \in(0,1)$is a predefined noise schedule controlling the step-wise noise scale, which controls the incremental noise intensity. The recursive formulation of the forward process can be expressed as:

Where is standard normal noise. We define the noise retention factor at step

*t* as $\alpha_{t} =1-\beta_{t}$, To characterize the cumulative effect of noise addition up to step *t*, we introduce the cumulative product: $\overline{\alpha}_{t} =\prod_{i=1}^{t} \alpha_{i}$. Using these definitions, the noisy sample

*x*_t_ at step *t* can be directly derived from *x*_0_ via:

Since the forward process is a linear Gaussian model, we can derive the marginal distribution of *x*_t_ given *x*_0_ in closed form. By recursively expanding the noise addition steps, we obtain:. Thus, the conditional distribution of *x*_t_ given *x_0_* is:

2. Reverse Generative Process

In the reverse generative process, the goal is to iteratively denoise from pure noise to recover the original data distribution *x*_0_. To achieve this, we learn a **parameterized model** $p_{\theta} (x_{t-1} \text{∣}x_{t} )$ to approximate the true reverse transition distribution$q(x_{t} \text{∣}x_{t-1} )$. Each step of the reverse process $p_{\theta} (x_{t-1} \text{∣}x_{t} )$is modeled as a Gaussian distribution:

$$p_{\theta} (x_{t-1 }\text{∣}x_{t} )=N(x_{t-1} ;\mu_{\theta} (x_{t} ,t),\Sigma_{\theta} (x_{t} ,t))$$

Where, the mean $\mu_{\theta} (x_{t} ,t)$and the covariance matrix $\Sigma_{\theta} (x_{t} ,t)$are parameterized by neural networks.

3. Variational subordinate and noise prediction loss

To train the model, we aim to minimize the discrepancy between the generated distribution

$p_{\theta} (x_{0 })$ and the true data distribution $q (x_{0 })$. This is typically achieved through variational inference, where we optimize a Variational Lower Bound (VLB). For practical training, we adopt a noise-prediction loss to simplify the optimization process. Let $\varepsilon_{\theta} (x_{t} ,t)$be a neural network that predicts the noise injected at timestep *t*. The loss function is defined as:

Where,. This loss function directly fits the noise, simplifying the optimization process. By minimizing this loss, we can train the model to approximate every step of the reverse diffusion process.

Part 7: The pathway to reduce the iteration count in the imagination based on the Spintronic device.

The 1000-step denoising iteration commonly adopted in standard diffusion models incurs excessive latency overhead, making it difficult to meet the deployment requirements of latency-sensitive real-time applications such as autonomous driving. To address this core bottleneck, we propose a systematic solution from two key aspects: algorithmic optimization and device-level performance enhancement.

(1) **Algorithmic Optimization**

In Figure R2, the core reason why standard diffusion models require 1000 or even more denoising iterations lies in the mainstream ϵ-prediction (noise prediction) or v-prediction (velocity prediction) paradigm widely used in these models. According to the manifold hypothesis, natural data lies on a low-dimensional manifold, while noise and noise-mixed quantities are inherently distributed across the full high-dimensional space. This makes the learning task of ϵ-/v-prediction extremely challenging: the model must rely on thousands of iterations to gradually correct errors to achieve ideal generation performance, and may even suffer from catastrophic failure in high-dimensional pixel space.

To tackle this bottleneck, the team led by Kaiming He at MIT proposed the x-prediction paradigm that directly predicts clean data in their paper Back to Basics: Let Denoising Generative Models Denoise published in late 2025. This paradigm allows the network to focus only on the low-dimensional effective information of clean data while filtering out noise, eliminating the need to learn the full high-dimensional noise distribution, thus fundamentally reducing the complexity of the denoising task. The Just image Transformers framework designed based on x-prediction can achieve high-quality denoising and generation performance on the high-resolution ImageNet dataset with only 50 denoising iterations, achieving a 20× reduction in the number of iterations compared to the standard 1000-step setting.

Figure S4 The Manifold Assumption hypothesizes that natural images lie on a low-dimensional manifold within the high dimensional pixel space. While a clean image x can be modeled as on-manifold, the noise ϵ or flow velocity v (e.g., v = x−ϵ) is inherently off-manifold. Training a neural network to predict a clean image (i.e., x-prediction) is fundamentally different from training it to predict noise or a noised quantity (i.e., ϵ/v-prediction)^[2]^.

(2) **Device-Level Optimization**

Co-based devices driven by Spin-Orbit Torque (SOT) have achieved magnetization switching at a speed of 1 ns^[3]^, which verifies that ferromagnetic systems have the fundamental capability to realize nanosecond-scale switching. Interlaced magnetic materials inherit the core advantages of antiferromagnetic materials, with intrinsic ultrafast spin dynamics characteristics, and are expected to drive nanosecond-scale magnetization switching in the ferromagnetic layer. In addition, the theoretical storage/switching speed of interlaced magnets can reach the picosecond scale, which is two to three orders of magnitude higher than that of existing commercial storage technologies such as ferromagnetic memory^[4]^. This can further reduce the hardware operation latency of a single denoising iteration, and forms a synergistic effect with algorithmic optimization to fully meet the stringent real-time requirements of autonomous driving scenarios.

Part 8: The comparisons between our generative performance and both software platforms and neuromorphic devices.

Table S1 The comparison between software-based model and emerging neuromorphic hardware platforms

| Type | Device Number | Noise Type | Network  Model | Write Current/Voltage | FID | Accuracy | year |
| --- | --- | --- | --- | --- | --- | --- | --- |
| Software^[5]^ | - | Gaussian | Diffusion | - | 3.17 | - | 2020 |
| Memristors^[6]^ | 1 | Sigmoid | RBM | 1.75 V | - | 90% on MNIST | 2024 |
| Ferroelectric transistors^[7]^ | 1 | Sigmoid | RBM | 2.8 V | - | 91.8% on MNIST | 2026 |
| SOT-MRAM^[8]^ | 1 | Sigmoid | RBM | 1.3 V | - | 93% on MNIST | 2024 |
| VCMA-MRAM^[9]^ | 8 | Gaussian | Diffusion | 2.4 V | 12.1 | - | 2025 |
| **This work** | 1 | Gaussian | Diffusion | 2.2e11  A/m^2^ | 1.98 | 90% on CIFAR-10 | 2026 |

In Table R1, most emerging neuromorphic devices adopt voltage-modulated stochastic writing to mimic Sigmoid random noise, and they implement generation tasks based on traditional restricted Boltzmann machines (RBM) networks. However, RBM has inherent limitations: it is constructed as a shallow two-layer structure and cannot support deep feature fitting; meanwhile, it is only suitable for basic feature learning and simple low-dimensional data generation, failing in complex practical scenarios.

The diffusion model, proposed in 2020, fundamentally overcomes the drawbacks of RBM. It adopts deep network architectures such as U-Net, and exhibits far stronger generation capability for high-dimensional complex data compared with shallow RBM structures. In 2025, researchers from the University of California realized hardware diffusion generation by using VCMA-MRAM to simulate Gaussian noise, while this solution suffers from excessive device overhead.

Our work achieves two improvements. Compared with the VCMA-MRAM scheme, our design reduces the total device count by 87%. In terms of generation quality, our model achieves approximately 1.60-fold performance improvement over the software baseline (the FID score is reduced from 3.17 to 1.98). These quantitative comparisons clearly demonstrate that our work goes far beyond a simple device demonstration and significantly strengthens the advancement and practicality of this research.

**Reference**

[1] Brown W F. Thermal fluctuations of a single-domain particle [J]. Physical Review, 1963, 130(5): 1677-1686.

[2] Li T, He K. Back to basics: Let denoising generative models denoise [J]. (arXiv:2511.13720 ).

[3] Thermally stable β-tungsten for spin–orbit torque magnetic random-access memory [J]. Nature Electronics, 2025, 8(9): 766-767.

[4] Dai J, Han L, Zhou Z, et al. Research progress and future prospects of altermagnets [J]. SCIENTIA SINICA Physica, Mechanica & Astronomica, 2026, 56(2): 227503.

[5] Ho J, Jain A, Abbeel P. Denoising diffusion probabilistic models [C]. International Conference on Neural Information Processing Systems (NeurIPS). Red Hook, NY, USA. 2020: 6840-6851.

[6] Kim J, Kim H, Chung M, et al. Stochastic photo-responsive memristive neuron for an in-sensor visual system based on restricted boltzmann machine [J]. Nanoscale Horizons, 2024, 9(8): 2248-2258.

[7] Deng H, Lu J, Fan Z, et al. Implementation of restricted boltzmann machine using ferroelectric-based stochastic neurons and deterministic synapses [J]. Journal of Applied Physics, 2026, 139(6): 064102.

[8] Li X, Wan C, Zhang R, et al. Restricted boltzmann machines implemented by spin–orbit torque magnetic tunnel junctions [J]. Nano Letters, 2024, 24(18): 5420-5428.

[9] Cheng Y, Shu Q, Lee A, et al. Voltage-controlled magnetoelectric devices for neuromorphic diffusion process [J]. Nature Communications, 2025, 16(1): 5022.
